# Online health information – what the newspapers tell their readers: a systematic content analysis

**DOI:** 10.1186/1471-2458-14-1316

**Published:** 2014-12-23

**Authors:** Brian A McCaw, Kieran J McGlade, James C McElnay

**Affiliations:** School of Pharmacy, Queen’s University Belfast, 97 Lisburn Road, Belfast, BT9 7BL Ireland; School of Medicine, Dentistry and Biomedical Sciences, Queen’s University Belfast, 97 Lisburn Road, Belfast, BT9 7BL Ireland

**Keywords:** Newspapers, Newspaper article, Internet, Health information, Online health information

## Abstract

**Background:**

This study investigated the nature of newspaper reporting about online health information in the UK and US. Internet users frequently search for health information online, although the accuracy of the information retrieved varies greatly and can be misleading. Newspapers have the potential to influence public health behaviours, but information has been lacking in relation to how newspapers portray online health information to their readers.

**Methods:**

The newspaper database Nexis^®^UK was searched for articles published from 2003 – 2012 relating to online health information. Systematic content analysis of articles published in the highest circulation newspapers in the UK and US was performed. A second researcher coded a 10% sample to establish inter-rater reliability of coding.

**Results:**

In total, 161 newspaper articles were included in the analysis. Publication was most frequent in 2003, 2008 and 2009, which coincided with global threats to public health. UK broadsheet newspapers were significantly more likely to cover online health information than UK tabloid newspapers (p = 0.04) and only one article was identified in US tabloid newspapers. Articles most frequently appeared in health sections. Among the 79 articles that linked online health information to specific diseases or health topics, diabetes was the most frequently mentioned disease, cancer the commonest group of diseases and sexual health the most frequent health topic. Articles portrayed benefits of obtaining online health information more frequently than risks. Quotations from health professionals portrayed mixed opinions regarding public access to online health information. 108 (67.1%) articles directed readers to specific health-related web sites. 135 (83.9%) articles were rated as having balanced judgement and 76 (47.2%) were judged as having excellent quality reporting. No difference was found in the quality of reporting between UK and US articles.

**Conclusions:**

Newspaper coverage of online health information was low during the 10-year period 2003 to 2012. Journalists tended to emphasise the benefits and understate the risks of online health information and the quality of reporting varied considerably. Newspapers directed readers to sources of online health information during global epidemics although, as most articles appeared in the health sections of broadsheet newspapers, coverage was limited to a relatively small readership.

**Electronic supplementary material:**

The online version of this article (doi:10.1186/1471-2458-14-1316) contains supplementary material, which is available to authorized users.

## Background

Approximately 7 in 10 adult Internet users in the UK and US search online for health information annually [1, 2]. Commonly reported motivators for seeking online health information include chronic illness [3, 4], self-diagnosis [2], receipt of a new diagnosis [5], dissatisfaction with health care providers [6] and searching for lifestyle advice [7].

While the Internet provides convenient public access to health information, evidence suggests that searching for health information is challenging for the average Internet user, not only due to the volume and variable quality of information that may be retrieved, but also due to differences in searching ability and comprehension among consumers [8, 9]. Furthermore, many studies have reported that the accuracy of health information retrieved in Internet searches varies greatly and can be misleading. For example, Agricola *et al.* reported recently that preconception advice retrieved via the Google search engine was generally inconsistent and frequently incomplete [10] and Singh *et al.* found that approximately one third of YouTube videos relating to rheumatoid arthritis contained misleading information and over 90% promoted unscientific therapies [11]. Using inaccurate or misleading health information for decision making purposes, without expert advice, could potentially have a serious negative impact on the individual user and on public health in general [12]. Thus, it is paramount that consumers are informed of the risks associated with searching for health information online and information seekers should be directed to accurate and credible web sites. Who should perform these roles? Media coverage is an important source of public knowledge on health-related issues and evidence suggests that the mass media has the potential to influence health behaviours [13].

Newspapers are an important element of the mass media and approximately one third of adults in Great Britain read at least one national daily newspaper each day [14]. However, evidence suggests that the quality of health reporting in newspapers tends to be poor. An evaluation of 500 health news stories published in US newspapers between 2006 and 2008 reported that between 62% and 77% of articles failed to adequately address costs, harms, benefits, the quality of the evidence and the existence of other options when covering health care products and procedures [15]. Furthermore, newspapers tend to overemphasise benefits and under-represent risks when reporting on health interventions [16, 17]. Nothing is known about how the newspaper media portray the Internet as a source of health information. Journalists often use health web sites as information sources for their articles but rarely comment on their quality or credibility [18]. Ideally, newspaper articles should be accurate and balanced so that readers can make informed decisions regarding the Internet as a source of health information. If newspaper reporting is inaccurate, imbalanced, or incomplete, readers may develop unrealistic perceptions of the value of online health information, therefore, the aim of the present study was to investigate how newspapers in the UK and US portray health information on the Internet, including social media, websites and blogs, to the public in terms of the frequency, nature and quality of reporting.

## Methods

### Study design

We employed systematic content analysis to examine how the highest circulation newspapers in the UK and US portrayed online health information in the 10-year period between 1^st^ January 2003 and the 31^st^ December 2012.

### Selection of newspaper articles

The Nexis^®^UK database is a full text archive of newspapers published globally and has been used widely in previous studies of media coverage of health-related issues [19–21]. We searched a purposive sample of UK newspapers (two Sunday newspapers and ten daily newspapers, together with their Sunday equivalents) with the highest circulation at the time of commencement of data collection (December 2012) [22]. This sample comprised *The Sun (The Sun on Sunday), Daily Mail (Mail on Sunday), Daily Mirror (The Sunday Mirror), Daily Star (Sunday Star), The Daily Telegraph (The Sunday Telegraph), The Daily Express (The Sunday Express), Daily Record (Sunday Record), The Times (The Sunday Times), The Guardian (The Observer), The Independent (Independent on Sunday), Financial Times, The i, The News of the World* and *The People.* Similarly, we searched the twelve highest circulation US newspapers [23], which comprised *USA Today, Wall Street Journal, The New York Times, Los Angeles Times, The Washington Post, The New York Post, New York Daily News, Chicago Tribune, Arizona Republic, Newsday, Houston Chronicle* and the *Denver Post.* All except *USA Today* and the *Wall Street Journal* publish daily and on Sundays.

### Search strategy

Following empiric testing of several search terms, we used the search term “Internet AND health information” to search the Nexis^®^UK database for all articles (including news articles, editorials, magazine supplements, letters, etc.) that contained any reference to the search term in either the headline or text during the period from 1^st^ January 2003 to 31^st^ December 2012. The primary researcher (BMcC) retrieved and read all of the archived newspaper articles. Items were excluded if online health information was mentioned only briefly (i.e. <10% of the article by word count), if they focussed on business issues (e.g. technology company share prices) or if online health information was mentioned only as part of an announcement, e.g. announcement of an adult learning class. We included only the article with the highest word count when an article was duplicated in both a daily newspaper and its Sunday equivalent. We searched the PubMed^®^ database using the same search terms and dates to compare the frequency of publication of newspaper articles with publication of scientific articles related to this topic.

### Article coding

We established an *a priori* coding system based on systems used in previously published systematic media content analyses [16–19]. This consisted of a codebook containing the list of variables to be researched, along with standardised responses and coding instructions, and a coding form (see Additional file 1). This approach provided a consistent coding framework and limited the potential for subjective judgement by coders. Two coders piloted the coding framework by coding a random sample of ten articles independently. Following the pilot, minor adjustments were made to the coding system to increase its specificity. The final coding frame comprised four main sections: firstly; the name of the newspaper, the title of the article, its year of publication and the newspaper section in which the article of interest appeared were recorded. Secondly; the themes covered, the perspective from which the article was written, whether the focus was on a particular health sector or illness, benefits/risks or barriers/facilitators relating to the use of online health information in routine clinical practice, and the source of the information contained in the article were noted. In the third section, coders were required to make subjective judgements on the main emphasis of the article, claim and quality of information. Finally, if a scientific journal article was identified as the source of information for the newspaper article, all reasonable steps were taken to obtain the scientific paper and its title, authors, publication date and disclosure of conflict of interest were recorded.

The primary researcher (BMcC) used the final coding form to manually code the selected articles. A second coder coded a 10% random sub-sample blindly and Cohen’s *kappa* (*κ*) scores were calculated to assess inter-rater agreement for questions with mutually exclusive answers. Questions with more than two answers were dichotomised, for example, “Type of benefits of health-related use of the Internet” (nine options provided), was collapsed to “Was a benefit stated?” (yes/no).

Where diseases were specifically mentioned, they were classified according to the relevant chapter in the British National Formulary (BNF), 63rd edition (British Medical Association and the Royal Pharmaceutical Society, 2012).

### Statistical analysis

Following data extraction, codes were entered into SPSS (version 19, SPSS Inc, USA) for analysis of trends and comparison of variables between countries. Descriptive statistics were used to summarise the data. The Mann-Whitney U test was used to test for differences between means of continuous variables. Differences in the reporting of categorical variables in UK and US articles were assessed using the Chi square test (χ^2^) or the Fisher’s Exact test, as appropriate. Statistical significance was set at 0.05.

## Results

**Figure 1:**
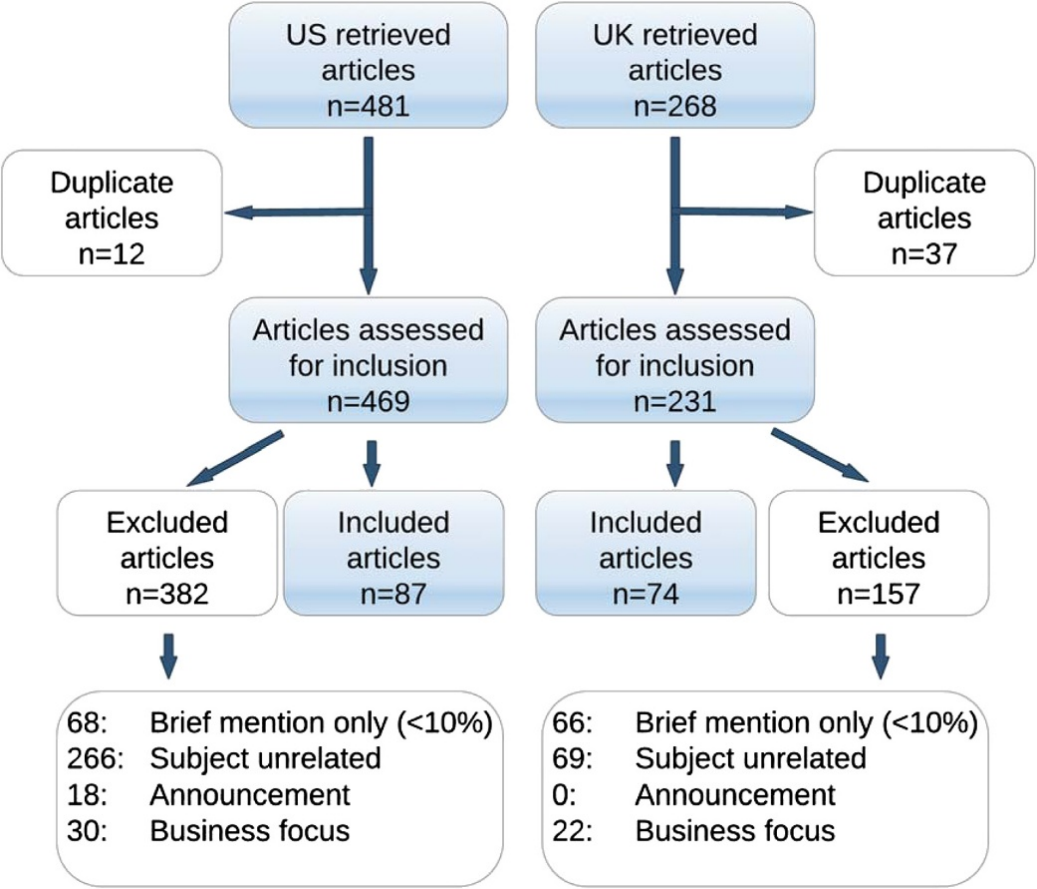
**Overview of the newspaper article selection process.**

Initially, 749 newspaper articles were retrieved, of which 161 articles, 74 from UK papers and 87 from US newspapers remained following removal of duplicates and excluded articles (Figure 1). Inter-rater *kappa* values ranged from 0.5 to 1.0, indicating moderate to perfect agreement between coders [24]. The mean inter-rater *kappa* value across all of the coded variables was 0.65; this is similar to the level of inter-rater agreement reported in previous quantitative content analyses involving the newspaper media [19, 25].

### Frequency of newspaper reporting

**Figure 2:**
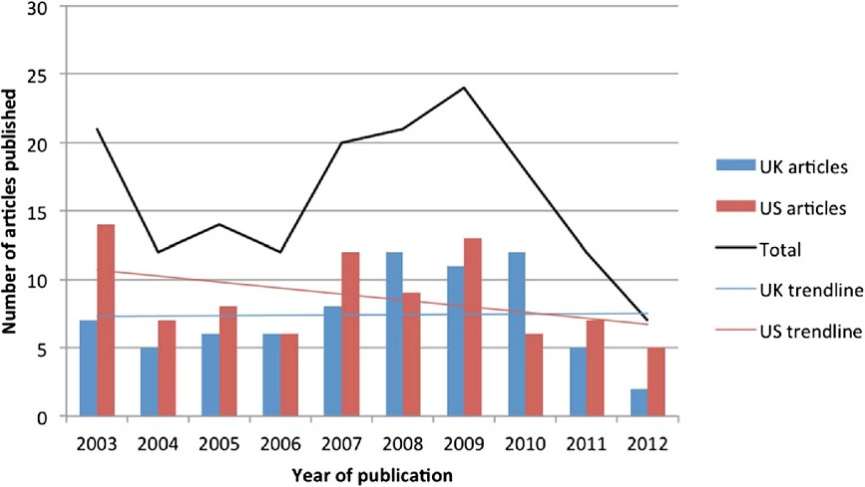
**Annual frequencies of relevant articles published in UK and US newspapers.**

**Figure 3:**
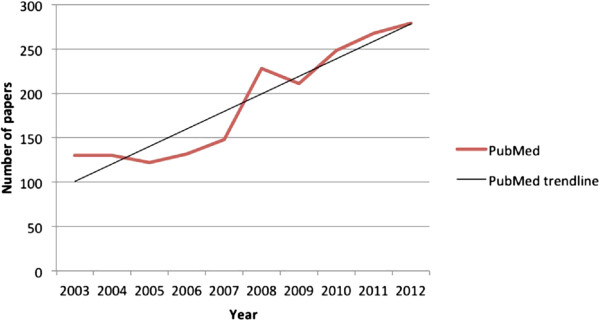
**Annual frequencies of scientific papers retrieved from the PubMed database using the search term “internet AND ‘health information’”.**

The number of articles retrieved from UK and US newspapers ranged from 7 in 2012 to 24 in 2009, indicating a low publication frequency relating to this topic. The highest numbers of articles were published in 2003 (21 articles), 2008 (21 articles) and 2009 (24 articles), with a marked decline in reporting on online health information after 2009 (Figure 2). In the UK, the overall trend in reporting remained relatively constant throughout the 10-year period, while an overall downward trend was observed in the US. During the same period there was an increase in scientific articles on this topic archived in PubMed^®^ (Figure 3), indicating that, during this time, online health information is a topic that has been researched actively.

### Newspaper type and positioning of articles

Strictly speaking, the terms ‘broadsheet’ and ‘tabloid’ refer to newspaper dimensions, however, broadsheet newspapers are perceived to be more intellectual in content in comparison to tabloids, which tend to report more sensationalist and celebrity material. Articles relating to online health information were published more frequently in ‘broadsheet’ newspapers than in ‘tabloid’ newspapers. Indeed, only one relevant article was found in the US tabloid press over the entire 10-year period of interest. In the UK, on average, 4.9 articles (SD 2.8) were published in broadsheet newspapers per year, which was significantly higher than the average of 2.5 (SD 2.0) articles published in tabloid newspapers annually (p = 0.04). In approximately two thirds (68.3%) of articles, it was obvious from the headline that the article related to health information on the Internet. Approximately a quarter of the selected articles (24.8%) were published in health sections and approximately one fifth appeared in feature (18.6%) and business (18.0%) sections. Interestingly, on only one occasion did the topic feature in the editorial/leader section, indicating the low priority given to the topic by newspaper editors.

### Authorship and information sources

Journalists wrote a substantial proportion of the articles (83.2%) and health professionals wrote relatively few (9.9%), although, in approximately a quarter (22.4%) of the articles authored by journalists, a health professional was cited as the main source of the information. Other sources included published reports or articles, or their authors (18.6%), spokespersons from the IT industry (12.4%) or from a Government/National Health Service (NHS) department (9.3%). Thirty articles were informed by a scientific report or journal article. The most frequently cited reports were those published by the Pew Internet and American Life Project. Almost two thirds of the articles (62.7%) included quotations from patients, medical or industry experts.

### Content of newspaper articles

Online health information was the main theme in the majority (65.2%) of articles. Other themes included the Internet as a medium for health-related communication between the public and/or health professionals (11.8%), access to online personal health records (8.7%), developments in Internet technology (5%) and online disease management tools (4.3%). The majority of articles (67.1%) mentioned or recommended specific web sites. In approximately one fifth (19.3%) of articles, the main focus was on the Internet as a channel for conveying health information in a public health context, for example, during the 2009 swine flu pandemic USA Today reported: “*Internet users have ramped up their searching, chatting and blogging of up-to-the-minute news on the symptoms and spread of swine flu since its sudden appearance this month. It's a trend health experts say is effective in rapidly pushing out public health information, using technology not available during the deadly, worldwide flu outbreaks of decades past*” (Gillum J. “People mine Net for everything flu; technology provides wealth of information – not all scientific”. USA Today. 29 April 2009; News, p7a).

**Figure 4:**
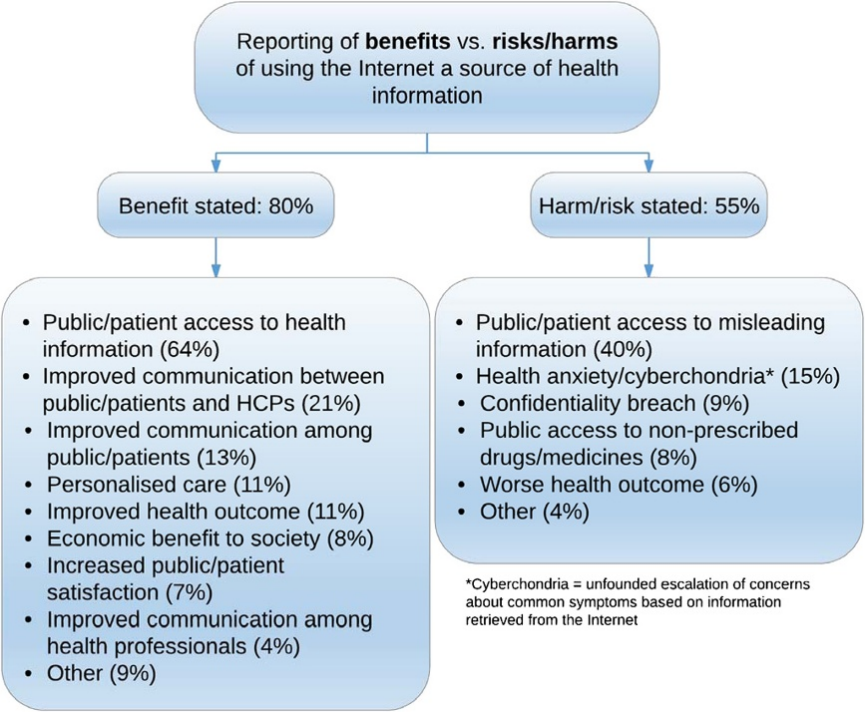
**Summary of reporting of benefits versus risks of online health information.**

Approximately half (49.1%) of the selected articles linked online health information to specific diseases, disease groups (e.g. cancer) or general health topics (e.g. women’s health). Using the BNF classification, the most frequently mentioned diseases related to the central nervous system (Table 1). Diabetes was the most frequently mentioned single disease, cancer the commonest group of diseases and sexual health was the most common general health topic. There was no significant difference between UK and US newspaper reporting in relation to the frequencies of mentioning diseases in each of the BNF classifications (p > 0.05). In addition, lifestyle issues, such as weight loss, alcohol consumption and exercise featured in approximately one fifth (19.2%) of the articles and ten articles focused on the Internet as an information source during pregnancy.Overall, 80% of articles mentioned benefits and 55% mentioned risks associated with health information on the Internet. Public access to health information was the most frequently reported benefit (64%) and access to misleading information was the most frequently cited risk (39.8%) (Figure 4). Most articles (41%) were written with a mixed slant, portraying benefits and risks equally. A slightly smaller proportion (38.5%) was positively slanted, i.e. mainly expressing benefits, and relatively few articles had a negative (11.2%) or neutral (9.3%) slant (i.e. no benefits or risks expressed). Interestingly, articles in US newspapers mentioned benefits more often than UK articles (81.6% vs. 77.0%) and risks less often (50.6% vs. 59.5%), although these differences were not significant (p > 0.05).

**Table 1:** **Classification of articles linking specific diseases with online health information**

BNF classification/topic	Number of articles
Central nervous system	51
Malignant disease	45
Cardiovascular disease	40
Infections	33
Endocrine	22
Obstetrics, gynae and urinary tract	12
Respiratory	11
Gastrointestinal	10
Musculoskeletal	6
Skin	6
Nutrition and blood	2
**Other health topics**	
Sexual health	6
Women’s health	4
Men’s health	4
Disability	3
Travel health	1
Sleep apnoea	1

**Figure 5:**
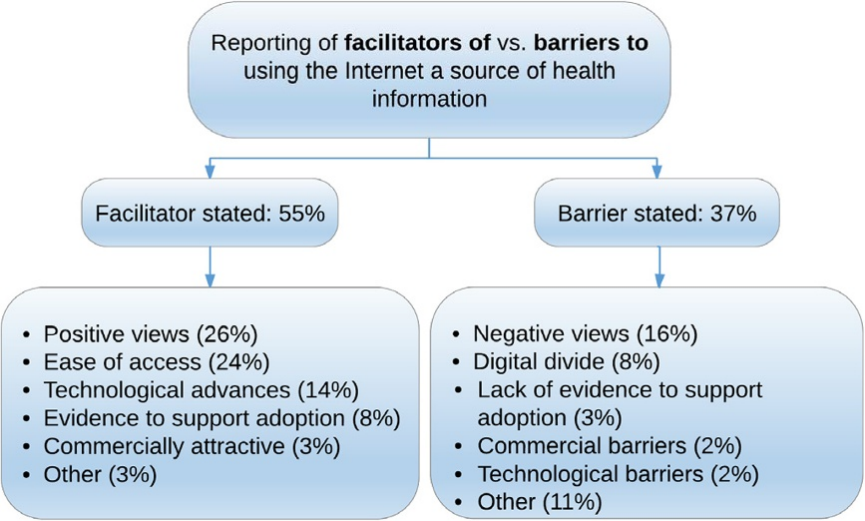
**Summary of reporting of barriers to versus facilitators of using online health information.**

There was no significant difference between UK and US newspapers in the frequency of reporting of facilitators and barriers to using online health information in routine clinical practice (p > 0.05). Facilitators were mentioned in 55.3% of articles (Figure 5); ease of Internet access and the expression of positive views by health professionals were the most frequently reported facilitators, for example “*We need to help them sort through it, not discourage the use of information. We have to acknowledge that patients do this research. It's important that instead of fighting against it, that we join them and become their coaches in the process”* (Parker-Pope, T. You’re sick. Now what? Knowledge is power. The New York Times. 30 September 2008; Science Desk, p1). Barriers were stated in 37.3% of articles (*kappa* = 0.5); the most frequently cited barrier was the negative viewpoint of health professionals *“Some doctors are less enthusiastic. People think all they need is some basic medical information and off they go. They even suggest that doctors could soon be out of a job"* (Bird J. ‘More like a conversation between equals’. The Financial Times. 27 June 2011; FT Health, p3).

### Balance and quality of newspaper reporting

The majority of articles (83.9%) were rated as having balanced judgement, i.e. the authors neither made exaggerated nor understated claims in comparison with the generally accepted status of online health information. The quality of information presented in each article was rated with the aid of descriptors on a scale of 1-10. Higher scores indicated higher quality reporting. A typical high quality article had balanced judgement, was based on evidence, and included quotations from subject experts, whereas, an article was rated as poor if it was anecdotal, lacked balanced judgement and did not include any evidence in support of its claims. Overall, 47.2% of the articles were rated as having excellent quality reporting (scored 8-10), 32.9% presented average/good quality information (scored 4-7) and 19.9% reported poor quality information (scored 1-3). We found no difference in the quality of reporting in UK articles compared to US articles (p > 0.05).

## Discussion

We found a low frequency of reporting on online health information in the highest circulation UK and US newspapers during the period 1^st^ January 2003 to 31^st^ December 2012. During the same period, the number of relevant research studies archived in PubMed^®^ more than doubled from 130 papers in 2003 to 279 papers in 2012. Newspapers are more likely to report on studies that have been press-released [26], therefore, the low level of reporting may be attributed to lack of promotion of research to newspaper editors by scientists or journals that publish in this area. Alternatively, newspaper editors may perceive that the use of the Internet as a health information source is not newsworthy or that the potential for harm associated with reliance on online health information is not an important public health issue.

Although overall UK and US newspaper reporting on online health information was low, peaks were evident in 2003 and in 2009 (Figure 2). During the analysis, we noted that the majority of articles published in these years reported the advice and information available online during the Severe Acute Respiratory Syndrome (SARS) outbreak in 2003 and the H1N1 influenza pandemic in 2009. This suggests that both UK and US newspaper editors saw a need to inform the public where to look for health information at times when public health was threatened. Indeed, UK newspaper reporting on the H1N1 virus in general peaked during the summer of 2009, mirroring the peak in UK cases of swine flu [25]. This finding supports Gupta and Sinhas’ assertion that coverage of health concerns in the news media tends to be higher when the issue affects the greatest number of people in their audience [27].

### Broadsheet versus tabloid reporting on online health information

A broad range of newspapers across the readership spectrum was included in the study. Articles in the US newspaper media were almost exclusively published in broadsheet newspapers. Similarly, significantly more UK articles were published in broadsheets even though tabloid papers made up a greater proportion of the UK sample. The National Readership Survey indicates that the three highest circulation newspapers in the UK (*The Sun, Daily Mail, Daily Mirror*) are all tabloids and are predominantly read by lower (C2DE) social classes [14]. Thus, readers of the tabloid press are unlikely to receive guidance on searching for or using online health information, or web site recommendations, from their newspapers. Also, within broadsheet newspapers, the majority of articles appeared in their ‘health’ sections, which suggests that these important messages may be reaching a very limited range of readers.

### Content of newspaper articles

Our results support Adelman and Verbrugge’s suggestion that diseases associated with high mortality rates receive the highest volume of newspaper coverage [28]. In articles that linked online health information to specific diseases, diabetes was the most frequently mentioned illness, while the most frequently mentioned disease categories were the central nervous system (CNS), malignant disease and cardiovascular disease. Articles that discussed online health information in relation to CNS disease encompassed a wide range of both neurological and mental illnesses, although depression was the most frequently mentioned disease in this category. The relatively high level of newspaper reporting on this illness correlates with suicide being the leading cause of death in adults under the age of 35 years in the UK [29].

Our results reinforce the suggestion of previous researchers that newspapers overemphasise benefits and under-represent risks when reporting on health interventions [16, 17]. It was interesting to observe that this disparity was greater among US newspapers, although the differences between UK and US newspapers were not statistically significant. Overstating the benefits of online health information may raise public expectations unrealistically, potentially leading to harm if an individual acts on misleading information without discussing their intentions with a health professional. The acceptability of the Internet as a credible source of health information in clinical practice largely depends on how it is perceived by health care professionals. Opinions expressed in articles were mixed although more professionals (55%) expressed positive views.

### Quality of press reporting

Our findings add to the body of evidence that the quality of newspaper reporting on health issues is variable. Less than half of articles were classified as having excellent information and the remainder were deemed to be of average/good or poor quality. Wilson *et al.* reported poor but improving quality of newspaper reporting on a variety of health interventions between 2004 and 2008 [30] whereas Hilton and Hunt found that newspaper reporting during the 2009 H1N1 influenza epidemic was ‘largely measured’ [25].

### Strengths and limitations of study

This is the first comprehensive investigation of how the highest circulation newspapers in the UK and US portray online health information to their readers. Although the mass media encompasses the Internet, television, radio, newspapers and magazines, we limited the scope of our study to the newspaper media for several reasons. Firstly, newspapers have a wide readership in both the UK and US. Secondly, the existence of an online database of full text newspaper articles provided an efficient mechanism to search for and obtain articles published within the period of interest. Thirdly, there is evidence of a strong correlation between newspaper reporting and other mass media coverage of similar issues [31]. Our analysis was limited to higher circulation newspapers, although, circulation figures are estimated based on the number of newspapers sold and not on the actual readership. Finally, there was limited availability of some US newspaper articles within the Nexis^®^UK database. Only the previous six months of *Los Angeles Times* articles and only abstracts of *Wall Street Journal* articles were available. Further limitations are the retrospective nature of the data collection, although a prospective study over 10 years would be impractical, and the use of a single coder for the majority of the data collection, although a calibration exercise with a second coder was undertaken.

## Conclusions

The extent of newspaper coverage of health information on the Internet was found to be low in comparison to the level of research published on this topic. In common with the findings of previous research on newspaper coverage of health issues, journalists tended to emphasise the benefits and understate the risks of online health information, and the quality of reporting varied considerably. Articles that reported on online health information focussed on common illnesses that are associated with high mortality rates. Nevertheless, newspaper editors perceived a need to report on online information when public health was threatened by global epidemics. Dissemination was generally via the health sections of broadsheet newspapers, limiting coverage to a relatively small and potentially already well-informed readership.

## Electronic supplementary material

Additional file 1: **Code framework.pdf – framework used to code newspaper articles.** (PDF 152 KB)
