# Supplementary material for: Online health information – what the newspapers tell their readers: a systematic content analysis
Source: BMC Public Health. 2014 Dec 23;14:1316. doi: 10.1186/1471-2458-14-1316 (PMC4326503; doi:10.1186/1471-2458-14-1316)
Supplement: Supplementary file 1 — Additional file 1: Code framework.pdf – framework used to code newspaper articles. (PDF 152 KB) [file 12889_2014_7480_MOESM1_ESM.pdf]

Online health information – what the newspapers tell their readers: a systematic content analysis

CODE BOOK

Coder ID: *Insert initials* **Article number:** *Insert article number*

CATEGORY AND OPTIONS	SPECIFY
**1. Basic information**	
**1.1 Name of newspaper**<br>1. The Sun<br>2. The Sun on Sunday<br>3. Daily Mail<br>4. Mail on Sunday<br>5. Daily Mirror<br>6. Sunday Mirror<br>7. Daily Star<br>8. Sunday Daily Star<br>9. Daily Telegraph<br>10. Sunday Telegraph<br>11. Daily Express<br>12. Sunday Express<br>13. Daily Record<br>14. Sunday Daily Record<br>15. The Times<br>16. Sunday Times<br>17. Financial Times<br>18. The Guardian<br>19. The Observer<br>20. The People<br>21. The i Paper<br>22. The Independent<br>23. Independent on Sunday<br>24. The News of the World<br>25. USA Today<br>26. Wall Street Journal<br>27. New York Times<br>28. Los Angeles Times<br>29. Washington Post<br>30. New York Post<br>31. New York Daily News<br>32. Chicago Tribune<br>33. Arizona Republic<br>34. Newsday<br>35. Houston Chronicle<br>36. Denver Post	*Select relevant newspaper*
**1.2 Year of publication**	*Insert year of publication*

**1.3 Section of newspaper**<br>0. Not specified<br>1. News<br>2. Editorial/leader<br>3. Feature<br>4. Letter<br>5. Business/financial<br>6. Health/Life<br>7. Magazine/supplement<br>8. Other (please specify...)	*Insert section of newspaper*
**2. Article content**	
**2.1. What is the main theme regarding use of the Internet in the article? (select one only)**<br>1. Information source<br>2. Communication tool<br>3. Disease specific info<br>4. Disease management<br>5. Online health records<br>6. Technology<br>7. Other (please specify...)	• *Select the* ***<u>main theme</u>*** *of the article:*<br>*1. Information source – the Internet is a health education / promotion / information source*<br>*2. Communication tool – facilitating communication between public / patients / Health professionals via blogs / forums / e-mail / social networking*<br>*3. Disease specific – article focuses on the Internet as an information source for patients with specific illness(es)*<br>*4. Disease management – self-management / remote monitoring / remote management / remote health-care delivery*<br>*5. Online health records – Internet as a repository for personal health records*<br>*6. Technology –article focuses on information technology / health apps*
**2.2. Other themes covered**<br>0. None<br>1. Yes (Please specify…)	• *Using the list in 2.1, indicate any other themes covered*
**2.3. Key perspective (select one only)**<br>1. Scientific<br>2. Political<br>3. Legal<br>4. Policy related<br>5. Societal<br>6. Industry<br>7. Economic<br>8. Others (please specify...)	• *Select the perspective from which the* ***<u>entire article</u>*** *is written:*<br>*1. Scientific – academic/research or survey report*<br>*2. Political – relating to party agenda/manifesto*<br>*3. Legal – relating to current/future legislation*<br>*4. Policy related – relating to Government policy*<br>*5. Societal – relating to impact on society*<br>*6. Industry – relating to the IT or health industries*<br>*7. Economic – relating to the UK or international economy*
**2.4. First mention of health-related use of the Internet**<br>1. Headline	• *Select where health-related use of the Internet is first mentioned:*<br>*1. Headline - it is obvious from the*

2. Body of text	*headline that the article relates to health-related use of the Internet (e.g. includes the word Internet, web, digital, online, app, etc.)*<br>*2. Body - it is not obvious from the headline that the article relates to health-related use of the Internet*
**2.5 Health sector (select one only)**<br>0. Not specified<br>1. Public health<br>2. Primary care<br>3. Secondary/Tertiary care<br>4. Other (please specify...)	• *Select the health sector that is the focus of the article:*<br>*0. Not specified - does not focus on a specific health sector*<br>*1. Public health – focuses on a public health issue or issues*<br>*2. Primary care - focuses on patients in the community or residential care*<br>*3. Secondary/Tertiary care - focuses on patients in general hospitals or specialist referral centres*
**2.6. Linked disease (or group of diseases)**<br>0. Not Specified<br>1. Cardiovascular<br>2. Central Nervous System<br>3. Respiratory<br>4. Gastrointestinal<br>5. Endocrine<br>6. Cancer<br>7. Infection<br>8. Immunological<br>9. Musculoskeletal<br>10. Other (please specify…)	• *Please indicate **all** diseases (or group(s) of diseases) mentioned (consult BNF for classification of disease)*
**2.7. Benefit/advantage of health-related use of the Internet**<br>0. Not mentioned (skip to 2.9)<br>1. Benefits stated<br>2. None (stated no benefit)	• *Please indicate if **any** benefits or advantages of health-related use of the Internet are stated in the article*
**2.8. Type of benefits of health-related use of the Internet**<br>1. Personalised care<br>2. Public/patient access to health information<br>3. Communication between public/patients and health professionals<br>4. Communication among public/patients<br>5. Communication among health professionals	• *Please indicate **all** potential benefits mentioned in article here.*

6. Improved health outcomes for public/patients<br>7. Public/patient satisfaction<br>8. Economic benefit to society<br>9. Others (please specify...)	
**2.9. Potential harm/risk of health-related use of the Internet**<br>0. Not mentioned (skip to 2.11)<br>1. Harm/risk stated<br>2. None (stated no harm/risk)	• *Please indicate if* ***any*** *harms/risks of health-related use of the Internet are stated in the article*
**2.10. Type of harm/risk of health-related use of the Internet**<br>1. Misleading information<br>2. Public access to non-prescribed drugs/medicines<br>3. Deterioration in health outcome<br>4. Confidentiality<br>5. Health anxiety / cyberchondria<br>6. Others (please specify...)	• *Please indicate* ***all*** *potential harm/risks mentioned in article here.*
**2.11. Barrier to use of the Internet in routine clinical practice stated**<br>0. Not specified<br>1. None (stated no barrier)<br>2. Commercial<br>3. Technology<br>4. Access<br>5. Negative beliefs<br>6. Lack of evidence<br>7. Others (please specify ...)	• *Select* ***all*** *barriers to incorporation of the Internet into routine clinical practice:*<br>*0. Not specified – article does not specify any barrier*<br>*1. None – article states that no barrier exists*<br>*2. Commercial – not economically viable*<br>*3. Technology - not sufficiently developed*<br>*4. Access – not easily accessed by potential users*<br>*5. Negative beliefs – not supported by public or healthcare professionals*<br>*6. Lack of research evidence to support adoption*
**2.12. Facilitator to use of the Internet in routine clinical practice stated**<br>0. Not specified<br>1. None (stated no facilitator)<br>2. Commercial<br>3. Technology<br>4. Access<br>5. Positive beliefs<br>6. Evidence to support adoption<br>7. Others (please specify ...)	• *Select* ***all*** *facilitators to support incorporation of the Internet into routine clinical practice:*<br>*0. Not specified – article does not specify any facilitator*<br>*1. None – article states that no facilitator exists*<br>*2. Commercial – economically attractive*<br>*3. Technology - sufficiently developed*<br>*4. Access – easily accessed by potential users*<br>*5. Positive beliefs – supported by public or healthcare professionals*<br>*6. Research evidence supports adoption*

**2.13. Who or what is cited as the main source of information for the newspaper article?**<br>0. Not mentioned<br>1. Scientific journal/paper/report or its authors<br>2. Scientific meeting or its attendees/organisers<br>3. Scientists in general<br>4. Health care professional(s)<br>5. Government/Department of Health/NHS spokesperson<br>6. IT industry spokesperson<br>7. Health/pharmaceutical industry spokesperson<br>8. Other (please specify...)	*Please indicate the main source of information for the article*
**2.14. Main voice of information**<br>1. IT industry spokesperson<br>2. Health/pharmaceutical industry spokesperson<br>3. Scientist/researcher academic spokesperson<br>4. Health care professional<br>5. Journalist or sub-editor<br>6. Others (please specify…)	*Please indicate the main voice of information in the article.*<br><br>*If the journalist is a HCP, please select HCP.*
**2.15. Are direct quotes about health-related use of the Internet used in article?**<br>0. No<br>1. Yes (Specify source(s))	*Please indicate if direct quotations relating to health-related use of the Internet are used in the article and who is quoted*
**2.16 Are any health-related web site(s) mentioned in article?**<br>0. None mentioned<br>1. Yes	• *Does the article mention specific web site(s)?*<br>*0. None mentioned – does not mention any specific health-related web site(s)*<br>*1. Specific health-related web site(s) mentioned*
**3. Judgement and rating**	
**3.1. Article slant about health-related use of the Internet**<br>1. Positive<br>2. Negative<br>3. Mixed<br>4. Neutral	• *Based on the stated benefits (2.7) and risks (2.9)*<br>*1. Positive – mainly benefits stated*<br>*2. Negative – mainly risks stated*<br>*3. Mixed – benefits and risks equally stated*<br>*4. Neutral – no benefits or risks stated*
**3.2. The main claim about health-related use of the Internet in newspaper article/**	• *Is the 'main message' of the article exaggerated, balanced or understated*

**presentation style**<br>1. Exaggerated judgement<br>2. Understated judgement<br>3. Balanced judgement	*compared with conventional knowledge on the topic?*
**3.3. Quality of information presented from a researcher perspective**<br>1. 1-3 (poor)<br>2. 4-7 (average/good)<br>3. 8-10 (excellent)	• *Please indicate a score between 1 and 10 based on the following:*<br>*1. 1-3 (poor) – anecdotal, not balanced, no evidence*<br>*2. 4-7 (average/good) – balanced article*<br>*3. 8-10 (excellent) – evidence-based, balanced, includes quotations*

If a scientific paper is mentioned in the newspaper article, please complete the following:

**4. Scientific article details**	*Please complete this table for each scientific paper mentioned in the newspaper article*
**4.1 Title**	
**4.2 Authors**	
**4.3 Journal**	
**4.4 Date of publication**	
**4.5. Conflict of interest**<br>0. Not mentioned<br>1. None (stated no conflict of interest)<br>2. Yes (conflict of interest specified)	• *Are any links with industry mentioned in the scientific article?*
**4.6 Coverage of links with industry in newspaper article**<br>0. Not mentioned<br>1. None (stated no links exist)<br>2. Yes (links specified)	• *Are any links with industry mentioned in the newspaper article?*
